# Region Adaptive Single Image Dehazing

**DOI:** 10.3390/e23111438

**Published:** 2021-10-30

**Authors:** Changwon Kim

**Affiliations:** Korean Intellectual Property Office, Daejeon 35208, Korea; cw10.kim@korea.kr; Tel.: +82-42-481-8712

**Keywords:** dehaze, dark channel prior, bright channel prior, Shannon’s entropy, texture probability

## Abstract

Image haze removal is essential in preprocessing for computer vision applications because outdoor images taken in adverse weather conditions such as fog or snow have poor visibility. This problem has been extensively studied in the literature, and the most popular technique is dark channel prior (*DCP*). However, dark channel prior tends to underestimate transmissions of bright areas or objects, which may cause color distortions during dehazing. This paper proposes a new single-image dehazing method that combines dark channel prior with bright channel prior in order to overcome the limitations of dark channel prior. A patch-based robust atmospheric light estimation was introduced in order to divide image into regions to which the *DCP* assumption and the *BCP* assumption are applied. Moreover, region adaptive haze control parameters are introduced in order to suppress the distortions in a flat and bright region and to increase the visibilities in a texture region. The flat and texture regions are expressed as probabilities by using local image entropy. The performance of the proposed method is evaluated by using synthetic and real data sets. Experimental results show that the proposed method outperforms the state-of-the-art image dehazing method both visually and numerically.

## 1. Introduction

Outdoor images and videos in hazy or cloudy weather conditions often suffer from the loss of details, decrease in contrast, and shifted chromaticity due to light scattering by atmospheric particles. This phenomenon affects the performance of subsequent high-level computer vision tasks, such as object detection and recognition. Therefore, improving image quality and enhancing system robustness in challenging weather conditions play a crucial role as a pre-processing step for broad application values [1].

This problem has been studied extensively in the literature with two main approaches: methods that use multiple images and methods that use only a single image.

Multi-image dehazing uses additional information about the scene, such as multiple images taken under diverse conditions [2,3,4], two or more images taken with different degrees of polarization [5,6], or geometric features of the scene [7], and infrared and visible images [8] in order to determine transmission and obtain haze-free images.

Compared to multi-image dehazing, a single image can only provide intensities of the three channels. Thus, additional priors or constraints are required for single image dehazing. Solutions for single image dehazing methods based on additional priors or constraints have been intensively developed in recent years.

Tan [9] proposed a dehazing method by maximizing local contrast based on the prior such that the contrast in a fogless image is higher than that in a foggy image. Fattal [10] estimated the medium of transmission by considering that there is no correlation between object surface shading and the transmission map. Moreover, he introduced a color line prior to the observation that pixels in small image patches typically exhibit a one-dimensional distribution in the RGB color space in [11]. Nishino et al. [12] estimated the scene albedo and depth by introducing a Bayesian probabilistic method. Tarel et al. [13] introduced a contrast-based enhancing approach in order to remove haze effects based on the assumption that the atmospheric veil function changed gently in the local region. Later, they introduced an additional constraint that much of the image could be assumed to be a flat road in order to better process the road image in [14]. Meng et al. [15] added a boundary constraint on the transmission function for single-image dehazing. In order to perform contrast enhancement, Ancuti et al. [16] implemented image dehazing based on multi-scale fusion. In [17], images with different exposure levels are extracted from a series of gamma corrections, and multi-exposure image fusion (MEF)-based adaptive restructuring was applied to each image patch in order to fuse into a haze-removed image. This study was further extended by Zhu et al. [18]. In order to guide the fusion process, they analyzed both global and local exposures to construct a per-pixel weight map. Zhu et al. [19] proposed a color attenuation prior to create a linear model for estimating the scene depth. Negru et al. [20] calculated atmospheric veils based on a mathematical model that accounted for changes in fog density with distance. Wang et al. [21] improved the color attenuation prior by estimating atmospheric light with a haze-line prior and replacing constant scattering coefficients with dynamic scattering coefficients. Duminil et al. [22] proposed a new prior for removing atmospheric veils based on a physical model considering that fog appears thicker near the horizon rather than closer to the camera. The Naka–Rushton function was used to modulate the atmospheric veil according to empirical observations on synthetic fog images. Berman et al. [23] proposed a non-local approach based on the assumption that colors of a haze-free image are well approximated by a few hundred distinct colors. Bui [24] proposed a color ellipsoid prior for dehazing where the dark channel prior was a special prior vector.

The Retinex theory has been widely applied in the field of single image dehazing. Xie et al. [25] used a multi-scale Retinex algorithm for the luminance component in order to acquire a transmission map and combined the haze image model with a dark channel prior to recover the haze-removed image. Gao et al. [26] proposed an enhancement MSRCR algorithm for color fog images based on an adaptive scale. Multi-scale images were obtained by weighting and performing local corrections for reflection component estimation. To take advantage of Retinex’s enhancements and address the lack of information about image scenarios, Wang et al. [27] proposed a single-image dehazing method based on atmospheric light scattering physical models and image brightness components by using multiscale Retinex with color restoration (MSRCR). Park et al. [28] estimated improved illuminance and reflectance by using the bright channel, which is estimated to control the amount of brightness enhancement by iteratively minimizing a varying Retinex-based energy function. Tang et al. [29] proposed night image dehazing by decomposing the atmospheric light image from the input image using Retinex theory and accurately estimating the point-by-point transmission map by using a Taylor series expansion, according to an approach based on dark channel prior.

With the availability of large-scale paired data and the success of the Convolutional Neural Network (CNN) in various image processing and computer vision tasks, learning-based dehazing methods have become popular in recent years. Tang et al. [30] used random forests to learn a mapping function between haze-relevant features and their true transmission in image patches. Ren et al. [31] proposed a multi-scale convolutional neural network to learn a mapping function between hazy images and corresponding transmission maps in a coarse-to-fine manner. Later on, they designed a network to learn confidence maps and proposed a fusion-based approach for haze removal in [32]. Cai et al. [33] adopted a deep convolutional neural network structure (four-layer) model that was specially designed to embody image dehazing. Li et al. [34] proposed an All-in-One Dehazing Network (AOD-Net) by reformulating the physical scattering model. Zhang and Patel [35] proposed a densely connected pyramid dehaze network that can examine scene depth and atmospheric light simultaneously. A Grid dehazing Network (GridNet) based on an attention mechanism for single image dehazing was proposed in [36]. Qu et al. [37] regarded the dehazing task as an image-to-image translation problem and designed an enhanced pix2pix dehazing network (EPDN) in order to generate clear results. 

Among the various single-image dehazing, the most popular is the dark channel prior [38]. Their prior is based on the observation that the minimum color components of local patches in haze-free images are usually very close to zero. *DCP* is a simple but effective approach in most cases, although it requires high computational complexity due to the soft matting algorithm and produces artifacts in bright areas. In order to improve computational efficiency, other approaches such as bilateral filtering [39], median filtering [40,41], edge-preserving filtering [42], and guided filtering [43] are used to optimize the transmission instead of soft matting. To reduce the distortions in a large area of the sky or a bright white object where the dark channel prior is invalid, sky detection-based methods [44,45,46,47,48,49] and white object detection-based method are proposed [50]. Jackson et al. [51] estimated the initial transmission map by modeling the scattering coefficients using Rayleigh scattering theory and dark channel prior. In addition, linear transformation, tolerance, and offset are proposed in order to consider that *DCP* values are not zero in bright regions [52,53,54,55]. Inspired by *DCP*, the bright channel prior (*BCP*) was proposed in [56]. It was assumed that most local patches which are not covered by dark objects in haze-free outdoor images contain some pixels that have very high intensities close to the upper limit in at least one color (RGB) channel. The dark channel prior and the bright channel prior are combined to calculate a transmission map in [57,58]. Zhang et al. [57] separated the hazy image into the two regions based on the *BCP* values and estimated a transmission map by jointly considering both priors in each region. Yutong et al. [58] proposed an adaptive bi-channel prior by combining the dark and bright channel priors and corrected the inaccurate estimation of transmission map and atmospheric light values for both white and black pixels that do not meet the assumptions of the bi-channel priors.

Several approaches have been proposed that consist of using superpixels for haze removal [58,59,60,61]. In the superpixel domain, Tan and Wang [59] obtained a transmission map and then improved the transmission map by using a Markov random field. Wang et al. [60] used the superpixel to estimate the transmission of sky and non-sky area in order to reduce halo artifacts around sharp edges and color distortion in sky area. In [58,61], the superpixel method was adopted instead of rectangular local patches to calculate the initial transmission map.

Image entropy was used for single-image dehazing either to compute transmission maps or to evaluate the resulting images. Park et al. [62] estimated the transmission map by an objective function, which comprises information fidelity and image entropy at non-overlapped sub-block regions. Ngo et al. [63] determined that hazy density is highly correlated with contrast energy, entropy, and sharpness and estimated the transmission map by utilizing an optimization of the objective function considering contrast energy, entropy, and sharpness. Salazar-Colores et al. [64] used local Shannon entropy to detect and segment a sky region in order to reduce artifacts and improve image recovery of the sky region. Image entropy was used as a qualitative metric to evaluate the quality of dehazed images in [49,65].

In this article, a region adaptive single image dehazing method is proposed to overcome the limitations of the *DCP*. The main contributions are as follows:

- Dark and bright channel priors are combined, and the combined priors are further improved by accurate estimation of atmospheric light and by introducing region adaptive control parameters.
- Patch-wise bright pixel selection and atmospheric light candidate scores calculated from dark channel and saturation values are introduced for an accurate estimation of atmospheric lights.
- A region adaptive control parameter for deciding whether to decrease or increase haze removal rate is proposed based on flat and non-flat region segmentations using local Shannon entropy.

As a result, the proposed method effectively restores haze-removed images while reducing an undesirable artifact in a bright area.

The rest of the paper is organized as follows. In Section 2, related works are briefly reviewed. In Section 3, the details of the proposed algorithm are described. In Section 4, the experimental results and analyses are presented. Finally, conclusions are provided in Section 5.

## 2. Related Work

**Figure 1:**
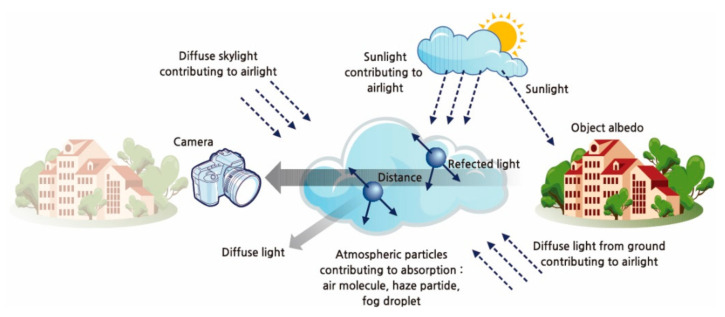
Atmospheric scattering model.

The atmospheric scattering model, which is shown in Figure 1, can be mathematically modeled as follows [1]:

(1) $$I\left(x\right)=t\left(x\right)J\left(x\right)+\left(1-t\left(x\right)\right)A,$$

where $I\left(x\right)$ is the hazy image, x is the spatial image index, $t\left(x\right)$ is the medium transmission map, $J\left(x\right)$ is the scene radiance, and A represents the global atmospheric light RGB vector. $t\left(x\right)$ is the transmission of scattered light in an homogeneous medium, which can be described as follows:

(2) $$t\left(x\right)=e^{-\beta d\left(x\right)},$$

where β is the scattering coefficient of the atmosphere, and $d\left(x\right)$ is the scene depth between the objects and the camera. Assuming β is constant, then $t\left(x\right)≅0$ when $d\left(x\right)\to \infty ,$ and $t\left(x\right)=1$ when $d\left(x\right)=0$.

Since the goal of image dehazing is to recover $J\left(x\right)$ from $I\left(x\right)$, $J\left(x\right)$ can be obtained by simply transforming Equation (1).

(3) $$J\left(x\right)=\frac{I\left(x\right)-A}{t\left(x\right)}+A$$

However, Equation (3) is an ill-posed problem because there are unknown variables $t\left(x\right)$ and *A*. Therefore, the performance of algorithms based on atmospheric scattering models depenSds on accurate calculations of *t(x)* and *A*.

### 2.1. Dark Channel Prior and Bright Channel Prior

Dark channel prior is based on the empirical investigation of the characteristics of clean outdoor images. It is observed that dark pixels have intensity values that are very close to zero for at least one-color channel within an image patch. The dark channel is defined as follows [38]:

(4) $$J^{d}\left(x\right)=\underset{y\in \Omega_{x}}{\min}\left(\underset{c\in \left\{R,G,B\right\}}{\min}J^{c}\left(y\right)\right)\;\to 0,$$

where $J^{c}$ is a color channel of J, and $\Omega_{x}$ is a local patch centered at x. From Equations (1) and (4), the transmission can be obtained by the following.

(5) $$t_{DCP}\left(x\right)=1-\underset{y\in \Omega_{x}}{\min}\left(\underset{c\in \left\{R,G,B\right\}}{\min}\frac{I^{c}\left(y\right)}{A^{c}}\right)$$

In order to be consistent with reality, a constant coefficient ω (0 < ω < 1) can be introduced into Equation (5) to keep some haze particles at a distance.

(6) $$t_{DCP}^{\omega }\left(x\right)=1-\omega \cdot \underset{y\in \Omega_{x}}{\min}\left(\underset{c\in \left\{R,G,B\right\}}{\min}\frac{I^{c}\left(y\right)}{A^{c}}\right)$$

In general, ω has a value of 0.9 to 0.95.

On the other hand, the bright channel prior assumes that most local patches and pixels in natural haze-free images contain some pixels that have intensities that are very high in at least one-color channel [56].

(7) $$J^{b}\left(x\right)=\underset{y\in \Omega_{x}}{\max}\left(\underset{c\in \left\{R,G,B\right\}}{\max}J^{c}\left(y\right)\right)\;\to 1.$$

The transmission of bright channel prior is calculated by combining Equations (1) and (7).

(8) $$t_{BCP}\left(x\right)=\frac{\underset{y\in \Omega_{x}}{\max}\left(\underset{c\in \left\{R,G,B\right\}}{\max}\frac{I^{c}\left(y\right)}{A^{c}}\right)-1}{\underset{c\in \left\{R,G,B\right\}}{\max}\frac{1}{A^{c}}-1}.$$

### 2.2. Air Light Estimation

#### 2.2.1. Dark Channel Prior

The atmospheric light A is calculated by choosing the highest intensity pixels from the top 0.1% brightest pixels in the dark channel in a haze image.

(9) $$A_{\infty }^{c}=I^{c}(arg\underset{y\in P_{0.1\%}}{\max}I^{d}\left(y\right))$$

In Equation (9), among the brightest pixels of 0.1%, the pixels corresponding to the maximum intensity in the color-channel of hazy input I are selected as the atmospheric light vector.

#### 2.2.2. Coarse-to-Fine Search Strategy

**Figure 2:**
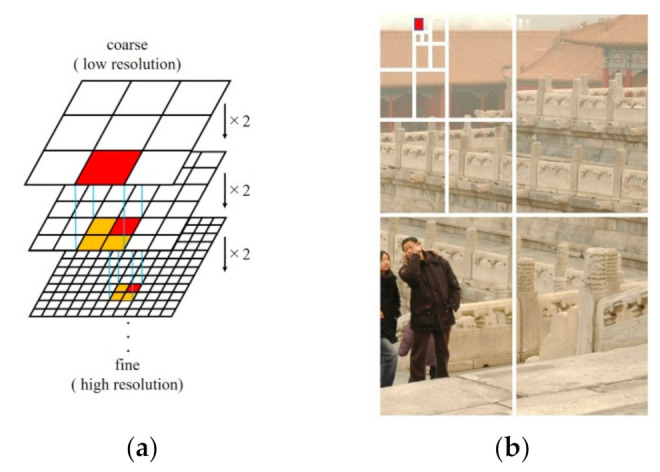
Atmospheric light estimation: (**a**) coarse-to-fine method and (**b**) quad decomposition method.

Iwamoto et al. [55] proposed a coarse-to-fine search strategy where they initially step down the resolution of the dark channel image and obtained the position of the brightest dark channSel value at the lowest resolution. Then, they recalculated the position of the largest dark channel value at the second lowest resolution and continued to recalculate the position of the brightest dark channel value until the original image size is reached. The schematic flow of the exploration strategy is shown in Figure 2a.

#### 2.2.3. Quad Decomposition Method

In the quad decomposition method proposed by Park et al. [62], the image is decomposed into quarters, and the quarter with the largest average luminance value is selected. The decomposition process is repeated on the selected quarter until the its size becomes smaller than a predetermined quarter size. The pixel with the smallest Euclidean norm relative to the white point in the RGB color space within the selected quarter is chosen as atmospheric light. The selected quarter is depicted in Figure 2b.

### 2.3. Shannon’s Entropy

**Figure 3:**
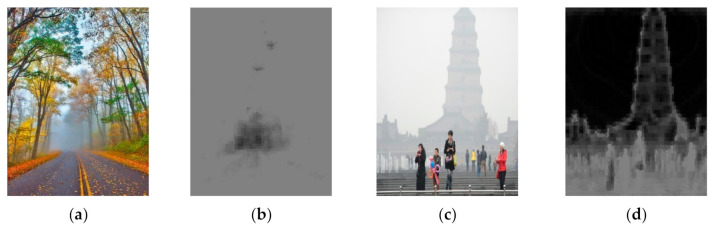
Examples of local image entropy: (**a**,**c**) input image, (**b**,**d**) local image entropy of (**a**,**c**).

Shannon entropy (Shannon, 1948) is originally proposed to quantify the information content in strings of text. It provides a measure of the amount of information required to describe a random variable. Similarly to the entropy concept widely used in information theory, when entropy is applied to a hazy image, high image entropy means that the image contains much detail, while low image entropy means that the image has less detail [63]. The local Shannon image entropy $E\left(x\right)$ on a local patch $\Omega_{x}$ is defined as follows:

(10) $$E\left(x\right)=-\sum_{y\in \Omega_{x}}^{L-1}p\left(y\right)\log p\left(y\right),$$

where *L* is the number of possible values for a pixel in $\Omega_{x}$ (in a grey-scale image L equals to 256), $p\left(y\right)=\frac{n_{j}}{N}$ is the probability that the grey-scale value j appears in $\Omega_{x}$, and $n_{j}$ is the number of pixels with the value j in $\Omega_{x}$. Figure 3 depicts the local image entropy of detailed and less detailed images. 

## 3. Proposed Method

This section describes the proposed image dehazing method in detail. In the proposed method, the superpixel method [66] is adopted instead of the rectangular local patch that is used in the existing *DCP*, and depth information of the scene is accurately expressed as an image patch. Firstly, a combined dark and bright channel prior is further improved by analyzing *DCP* and *BCP* in Section 3.1, and atmospheric light is estimated from the selected superpixels by using the newly proposed candidate scores in Section 3.2. In Section 3.3, a region adaptive haze control parameter designed to prevent artifacts in the hazy and bright regions with less detail and to improve the haze removal rate in the hazy region with a lot of detail is introduced based on the flat map calculated from Shannon entropy. Finally, transmission, *t(x)*, is calculated by using the region adaptive haze control parameter and refined in order to obtain a haze-free image.

### 3.1. Modified Dark and Bright Channel Prior

One of the reasons of the side effects of conventional *DCP* and *BCP* is that the characteristics of transmission function are overlooked. From Equation (3), transmission can be directly calculated as follows.

(11) $$t\left(x\right)=\frac{I\left(x\right)-A}{J\left(x\right)-A}\;$$

Since $0\leq t\left(x\right)\;\leq 1$, given the global atmospheric light *A*, the hazy input image can be separated into two parts.

(12) $$\begin{array}{ccc} \begin{array}{c} I\left(x\right)>A\\ I\left(x\right)<A \end{array} & ⇋ & \begin{array}{c} J\left(x\right)>A\to \;J\left(x\right)>I\left(x\right)\\ J\left(x\right)<A\to \;J\left(x\right)<I\left(x\right) \end{array} \end{array}\;$$

From Equation (12), it can be clearly observed that assumptions on *DCP* are valid only in the region where *I(x)* < *A*, and assumptions on *BCP* are valid in the region where *I(x)* > *A*.

This is observed by Zhang et al. [44], and they separated the input image into the two regions based on the *BCP* values. However, for $I^{b}\left(x\right)>A$ and $I^{d}\left(x\right)<A$, where $I^{b}\left(x\right)\;=\;\underset{y\in \Omega_{x}}{\max}\left(\underset{c\in \left\{R,G,B\right\}}{\max}I^{c}\left(y\right)\right)\;$ and $I^{d}\left(x\right)\;=\;\underset{y\in \Omega_{x}}{\min}\left(\underset{c\in \left\{R,G,B\right\}}{\min}I^{c}\left(y\right)\right)\;$, both *BCP* and *DCP* are valid. Thus, in order to consider the above case, the input hazy image should be separated into three regions instead of two. Then, Equation (11) can be rewritten as follows:

(13) $$t\left(x\right)=\left\{\begin{array}{cc} t_{BCP}\left(x\right)=\frac{A^{M}}{1-A^{M}}\cdot (I_{A}^{b}\left(x\right)-1) & I_{A}^{d}\left(x\right)>1\\ t_{DCP}\left(x\right)=\;1-I_{A}^{d}\left(x\right) & I_{A}^{b}\left(x\right)<1\\ 0.5\times \max\left(t_{BCP}\left(x\right),t_{0}\right)+0.5\times \max\left(t_{DCP}\left(x\right),t_{0}\right) & otherwise \end{array}\right.,$$

where $I_{A}^{d}\left(x\right)\;=\;\underset{y\in \Omega_{x}}{\min}\left(\underset{c\in \left\{R,G,B\right\}}{\min}\frac{I^{c}\left(y\right)}{A^{c}}\right)\;$, $I_{A}^{b}\left(x\right)\;=\;\underset{y\in \Omega_{x}}{\max}\left(\underset{c\in \left\{R,G,B\right\}}{\max}\frac{I^{c}\left(y\right)}{A^{c}}\right)\;$, and $A^{M}\;=\;\underset{c\in \left\{R,G,B\right\}}{\max}A^{c}$.

**Figure 4:**
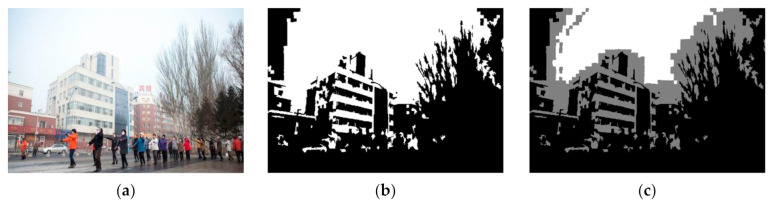
Region separation based on the atmospheric light A: (**a**) input image, (**b**) the result of Equation (11), and (**c**) proposed method.

Figure 4 shows that many regions belonging to *BCP* (white region) in Equation (13) satisfy both *BCP* and *DCP* (gray region). As observed from Equation (13), the estimation of A becomes more important because it is necessary to divide the region based on A and to apply an appropriate prior to each divided region. Therefore, in this paper, a novel atmospheric light estimation method will be proposed in Section 3.2. 

The oversimplified assumption explains another reason for the side effects of *DCP* and *BCP*. The original transmission of *DCP* and *BCP* $t_{actual}\left(x\right)$ is expressed as follows:

(14) $$t_{actual}\left(x\right)=\left\{\begin{array}{c} \frac{1-I_{A}^{d}\left(x\right)}{1-J_{A}^{d}\left(x\right)}\;\geq \;\;1-I_{A}^{d}\left(x\right)\\ \frac{I_{A}^{b}\left(x\right)-1}{J_{A}^{b}\left(x\right)-1}\;\geq \;A_{0}\cdot (I_{A}^{b}\left(x\right)-1) \end{array}\right.,$$

where $A_{0}=\frac{A^{M}}{1-A^{M}}$. For the areas that do not satisfy the dark channel prior, the value of the dark channel cannot be approximated to 0; similarly, for the areas that do not satisfy the bright channel prior, the value of the bright channel cannot be approximated to 1. Thus, $t_{actual}\left(x\right)$ is always greater than transmission $t\left(x\right),$ which is calculated based on the dark channel and bright channel priors. 

Inspired by the constant factor ω for controlling the haze removal rate in *DCP*, a region adaptive haze control parameter is introduced in order to consider the oversimplified assumption of *DCP* and *BCP*. Based on the Equation (14), modified transmission $t_{modified}\left(x\right)$ can be simply expressed as follows:

(15) $$t_{modified}\left(x\right)=\left\{\begin{array}{cc} \begin{array}{c} t_{DCP}^{modified}\left(x\right)=1-\omega_{p}\left(x\right)\cdot I_{A}^{d}\left(x\right)\geq \;\;t_{DCP}\left(x\right)\\ t_{BCP}^{modified}\left(x\right)=A_{0}\cdot \omega_{p}\left(x\right)\cdot (I_{A}^{b}\left(x\right)-1)+\left(1-\omega_{p}\left(x\right)\right)\;\geq \;t_{BCP}\left(x\right) \end{array} & 0\leq \omega_{p}\left(x\right)\leq 1, \end{array}\right.$$

where $\omega_{p}\left(x\right)$ is a proposed region adaptive haze control parameter. The equation for *BCP* is simply derived based on the assumption that $t_{modified}\left(x\right)\;=\;$ $1-\omega_{p}\left(x\right)$ when $I_{A}^{d}\left(x\right)\;=\;I_{A}^{b}\left(x\right)\;=\;1$. By combining Equations (13) and (15), the proposed transmission $t_{proposed}\left(x\right)$ is expressed as follows.

(16) $$t_{proposed}\left(x\right)=\left\{\begin{array}{cc} t_{BCP}^{modified}\left(x\right)t_{BCP}^{modified}\left(x\right)=A_{0}\cdot \omega_{p}\left(x\right)\cdot (I_{A}^{b}\left(x\right)-1)+\left(1-\omega_{p}\left(x\right)\right) & I_{A}^{d}\left(x\right)>1\\ t_{DCP}^{modified}\left(x\right)=1-\omega_{p}\left(x\right)\cdot I_{A}^{d}\left(x\right) & I_{A}^{b}\left(x\right)<1\\ \frac{\max\left(t_{BCP}^{modified}\left(x\right),1-\omega_{p}\left(x\right)\right)}{2}+\frac{\max\left(t_{DCP}^{modified}\left(x\right),1-\omega_{p}\left(x\right)\right)}{2} & otherwise \end{array}\right.$$

The details of region adaptive haze control parameter will be explained in Section 3.3.

### 3.2. Atmospheric Light Estimation

Due to the fact that most existing methods estimate atmospheric light by considering only brightness, white or bright landscape objects are often incorrectly chosen as atmospheric light. In the ideal case, if *d(x)* is large enough, *t(x)* tends to be very small according to Equation (2), and *I(x)* will be approximately *A*. Therefore, atmospheric light *A* can be estimated from deeper-depth regions. Since the depth of the scene is assumed to be positively correlated with haze concentration, atmospheric light candidate areas can be calculated by using the relationship between haze and contrast, saturation, and dark channel values [67]. Using the above relationship, the atmospheric light area candidate score is calculated as follows:

(17) $$Score_{A}\left(x\right)=(1-P_{s}\left(x\right))\cdot C\left(x\right)\cdot \left(1-S\left(x\right)\right)\cdot I^{d}\left(x\right),$$

where $S\left(x\right)$ and $I^{d}\left(x\right)$ denote saturation and dark channel value in the superpixel. Moreover, the portion of overexposed pixels in a superpixel $P_{s}\left(x\right)$ is introduced in order to avoid selecting overexposed pixels as candidates for ambient light. Candidate superpixels for atmospheric light estimation will be selected based on the score. The contrast related value $C\left(x\right)$ is defined as follows:

(18) $$C\left(x\right)=\left\{\begin{array}{cc} 1-\frac{\sigma_{x}}{\sigma } & \sigma_{x}<\sigma \\ 0 & \sigma_{x}\geq \sigma \end{array}\right.,$$

where $\sigma_{x}$ and σ denote a standard deviation of local patch $\Omega_{x}$ and entire image, respectively.

Inspired by the patch selection method for calculating color constancy [68], the hazy image is evenly divided into multiple rectangular patches (e.g., a 2 × 3 patch is used, but it is not limited). For each patch $F_{i,j}$, the mean of dark channel value is calculated as follows:

(19) $$\bar{D}\left(F_{i,j}\right)=\sum_{x\;\in F_{i,j}}\frac{I^{d}\left(x\right)}{N_{i,j}},$$

where $N_{i,j}$ is number of superpixels in patch $F_{i,j}$. 

**Figure 5:**
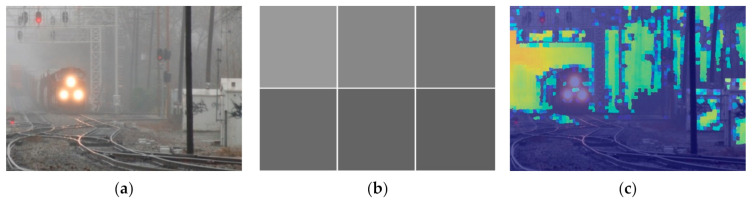
Atmospheric light estimation: (**a**) input image, (**b**) patch means of dark channel, and (**c**) candidate scores for each superpixels.

**Figure 6:**
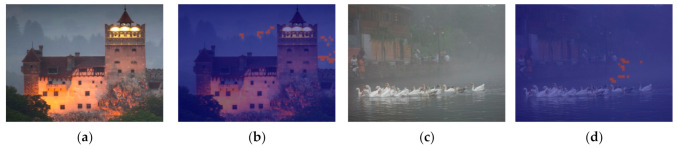
Selected superpixels for estimating atmospheric light: (**a**,**c**) input image, (**b**,**d**) selected superpixels.

The number of superpixels selected form patch $F_{i,j}$ is set to be proportional to the mean of the dark channel value $\bar{D}\left(F_{i,j}\right)$:

(20) $$N_{s}\left(F_{i,j}\right)=\left\{\begin{array}{cc} \frac{\bar{D}\left(F_{i,j}\right)}{\mu_{d}}N\varepsilon_{s} & ,\;\;\bar{D}\left(F_{i,j}\right)\geq \mu_{d}\\ 0 & ,\;\;\bar{D}\left(F_{i,j}\right)<\mu_{d} \end{array}\right.,$$

where $\mu_{d}$ is mean of the dark channel value of image, N is the total number of superpixes in the image, and $\varepsilon_{s}$ is a constant that determines the selecting rate, which generally takes a value of 0.001. An example of patch means of dark channel and candidate scores for each superpixels can be seen in Figure 5. Finally, the highest score $N_{s}\left(F_{i,j}\right)$ superpixels in patch $F_{i,j}$ are selected, and atmospheric light A is calculated by averaging the selected superpixels. The selected superpixels for calculating atmospheric light are shown in Figure 6.

### 3.3. Region Adaptive Haze Control Parameter

**Figure 7:**
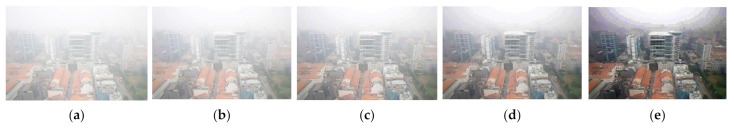
The effects of haze control parameter ω: (**a**) input hazy image, (**b**) recovered image (ω = 0.25), (**c**) recovered image (ω = 0.5), (**d**) recovered image (ω = 0.75), and (**e**) recovered image (ω = 1.0).

The effects of haze control parameter ω are shown in Figure 7. As ω increases, more haze is removed, and the visibilities are improved, but unwanted artifacts are produced in the sky area. As discussed in Section 3.1, the artifacts are caused by oversimplified assumptions of *DCP* and *BCP*; thus, region adaptive haze control parameters are introduced to address this problem. The region adaptive control parameter should improve visibility in high detail hazy areas while avoiding artifacts in low detail bright and hazy region such as sky areas. To this end, the region with and without detail should be determined first. In this paper, the area with detail is regarded as texture, and the area without detail is regarded as a flat.

#### 3.3.1. Texture and Flat Area Detection

**Figure 8:**
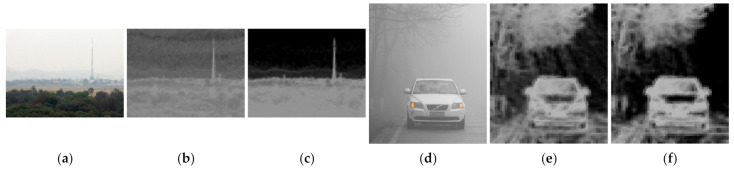
Comparison of local image entropy: (**a**,**d**) input image, (**b**,**e**) entropy calculated from input image, and (**c**,**f**) entropy calculated from gradient of image.

As mentioned in Section 2.3, image entropy can be used to characterize the texture of an image and to determine the amount of image information [69]. However, images affected by haze tend to have low image entropy values due to the biased distribution of brightness. This makes it difficult to distinguish between texture and flat areas. Therefore, instead of using an image, the gradient of the image is used to calculate entropy. As observed in Figure 8, entropy calculated from the gradient of the image has a larger difference in value than the entropy calculated from the image in the flat region and the texture region.

**Figure 9:**
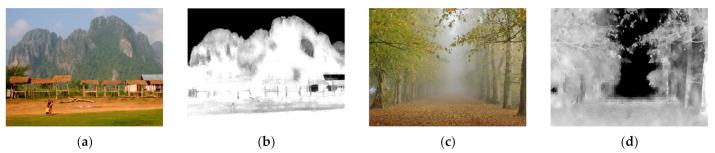
Examples of texture probability: (**a**,**c**) input image, (**b**,**d**) texture probability.

Local image entropy $E_{G}\left(x\right)$ is computed over the gradient image, and then the texture probability $P_{T}\left(x\right)$ is calculated as follows:

(21) $$P_{T}\left(x\right)=\left\{\begin{array}{cc} 1 & E_{G}\left(x\right)>T_{T}\\ \frac{E_{G}\left(x\right)-T_{F}}{T_{T}-T_{F}} & T_{F}<E_{G}\left(x\right)<T_{T}\\ 0 & E_{G}\left(x\right)<T_{F} \end{array}\right.,$$

where $T_{F}$ is the threshold obtained from OTSU threshold [52] of $E_{G}\left(x\right)$, and $T_{T}=T_{F}+64$. A high $P_{T}\left(x\right)$ value means a texture region, and a low $P_{T}\left(x\right)$ value means a flat region. An example of texture probability $P_{T}\left(x\right)$ can be seen in Figure 9.

#### 3.3.2. Region Adaptive Haze Control Parameter Calculation

In order to avoid artifacts in low-detail bright and hazy region, a prevention weight is introduced. The texture probability $P_{T}\left(x\right)$ has low values in the low detail region, and hazy image has low saturation values due to scattering and diffusion of reflected light in the atmosphere [67], and the dark channel value $I^{d}\left(x\right)$ has a high value at bright region. Based on the above relationship, the prevention weight for avoiding artifacts in low-detail bright and hazy region is defined as follows.

(22) $$W_{p}\left(x\right)=(1-P_{T}\left(x\right))\cdot \left(1-S\left(x\right)\right)\cdot I^{d}\left(x\right)$$

**Figure 10:**
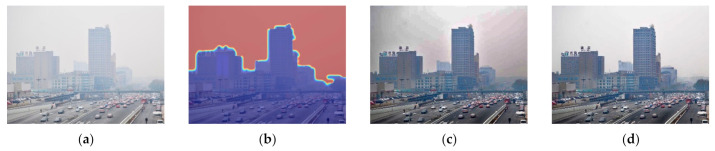
The effect of prevention weight: (**a**) input image, (**b**) prevention weight, (**c**) result without prevention weight, and (**d**) result with prevention weight.

The value of $W_{p}\left(x\right)$ becomes higher in bright and hazy regions with low detail and lower in regions where it is not. Figure 10 illustrates the prevention weight and shows the artifact suppression performance in low-detail bright and hazy regions. 

**Figure 11:**
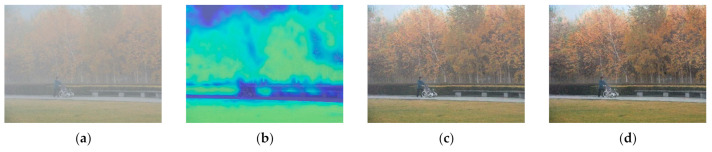
The effect of enhancement weight: (**a**) input image, (**b**) enhancement weight, (**c**) result without enhancement weight, and (**d**) result with enhancement weight.

In order to improve visibilities in high-detail hazy areas, an enhancement weight is introduced. Texture probability and local variance are high in regions with high detail, low saturation values in regions of hazy and enhancement weight should be inversely proportional to the prevention weight. By using the above relationship, the enhancement weight for improving visibilities in high-detail hazy areas is defined as follows:

(23) $$W_{E}\left(x\right)=\left(1-W_{p}\left(x\right)\right)\cdot P_{T}\left(x\right)\cdot D\left(x\right)\cdot \left(1-S\left(x\right)\right),$$

where $D\left(x\right)=\frac{\left\|I\left(x\right)-\mu_{x}\right\|}{N_{x}}$ is a variance of local patch $\Omega_{x}$. The value of $W_{E}\left(x\right)$ becomes lower in areas with high prevention weight $W_{p}\left(x\right)$ or low-detail and higher in high-detail hazy region. Figure 11 depicts the enhancement weight and shows the haze removal performance in high-detail hazy regions.

As inferred from Figure 7, a decrease in the value of $\omega_{p}\left(x\right)$ leaves a haze, and an increase in the value of $\omega_{p}\left(x\right)$ removes the haze, but artifacts may occur. Thus, $\omega_{p}\left(x\right)$ should be low when $W_{p}\left(x\right)$ is high and should increase when $W_{E}\left(x\right)$ is high. By using the above relation, $\omega_{p}\left(x\right)$ can be expressed as follows:

(24) $$\omega_{p}\left(x\right)=\omega_{0}\left(1-\omega_{p}W_{p}\left(x\right)+\omega_{E}W_{E}\left(x\right)\right),$$

where $\omega_{0}$ is a parameter for controlling the overall haze removal rate, and $\omega_{p}$ and $\omega_{E}$ are parameters for controlling the amount of prevention and enhancement.

### 3.4. Haze-Free Image Recovery

The patch level transmission $t_{proposed}\left(x\right)$ should be refined to contain pixel level transmission $t\left(p,\;q\right)$ using a guided filter [43]. With atmospheric light A and the refined transmission map $t\left(p,\;q\right)$, the haze-free image J can be recovered as follows.

(25) $$J\left(p,\;q\right)=\frac{I\left(p,\;q\right)-A}{\max\left(t\left(p,\;q\right),0.05\right)}+A$$

The transmission is limited by a lower bound (0.05), which is the same empirical value in [38], in order to avoid excessive enhancement. 

## 4. Experimental Results

In this section, the effectiveness of the proposed method is evaluated and verified qualitatively and quantitatively with the conventional method. The hazy images used in the experiment are divided into those with and without ground truth. The hazy images that have ground truth are collected from I-HAZE database [70], O-HAZE database [71], Synthetic Objective Testing Set (SOTS), and Hybrid Subjective Testing Set (HSTS) from a Realistic Single Image Dehazing (RESIDE) dataset [72]. On the other hand, real-world hazy images do not have ground truth images, but in order to check whether the proposed method is applicable to real life, real-world hazy images should be considered. In order to acquire a wide range of images for testing, 200 real-world hazy images were collected from the Realistic Single Image Dehazing (RESIDE) dataset. The proposed method was tested on hazy images with ground truth and real-world hazy images and compared with the He et al. [38], Meng et al. [15], Berman et al. [23], Zhu et al. [19], Ren et al. [31], and Cai et al. [33].

### 4.1. Quantitative Analysis

#### 4.1.1. Quantitative Comparison of Hazy Image with Ground Truth

In this subsection, the performance of dehazing algorithms was compared by using various hazy image sets containing ground-truth (haze-free) images. The performance of the algorithms can be evaluated by analyzing the similarity between the dehazing results and the ground-truth images by using the full reference metrics PSNR and SSIM. 

In Table 1, PSNR and SSIM values are calculated for images restored from I-HAZE, O-HAZE, SOTS indoor and outdoor, and HSTS datasets. In general, deep learning-based methods (i.e., Ren et al. and Cai et al.) show good numerical results for hazy images with ground truth. The proposed method showed the best or second highest performance in SSIM and PSNR, respectively. The quantitative metrics show that the proposed method effectively removes haze.

#### 4.1.2. Quantitative Comparison of Hazy Image without Ground Truth 

Since there is no reference image in the natural images experiment, the IQA evaluation index and image entropy (IE) were used to evaluate the quality of the dehazing results. As an IQA evaluation index, the natural image quality evaluator (NIQE) [73], the blind/referenceless image spatial quality evaluator (BRISQUE) which outputs the value range [0, 100] [74], and the perception-based image quality evaluation (PIQE) [75] are used in this paper. Moreover, image entropy (IE) describes the randomness distribution of the image, and its value denotes the amount of image information [62]. The better the picture quality, the smaller the NIQE, BRISQUE, and PIQE values and the higher the IE.

Table 2 shows the haze removal performance of natural images in NIQE, BRISQUE, PIQE, and IE. It can be observed that the proposed method outperforms other conventional methods except NIQE. This shows the effectiveness of the proposed method in a real case.

### 4.2. Qualitative Comparison

#### 4.2.1. Qualitative Comparison of Hazy Image with Ground Truth

**Figure 12:**
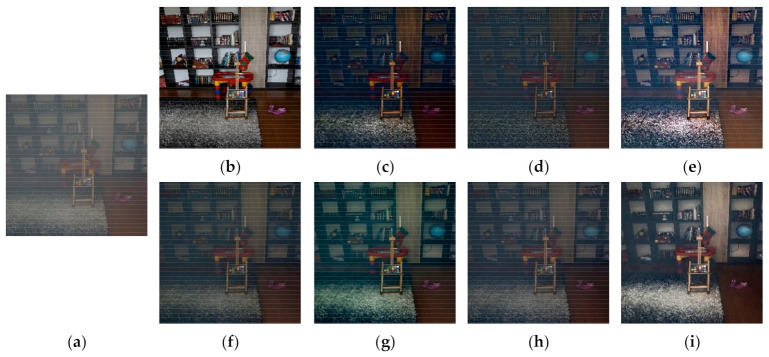
Comparison of dehazing results from I-HAZE dataset: (**a**) hazy image, (**b**) ground truth, (**c**) He et al., (**d**) Meng et al., (**e**) Berman et al., (**f**) Zhu et al., (**g**) Ren et al., (**h**) Cai et al., and (**i**) proposed.

**Figure 13:**
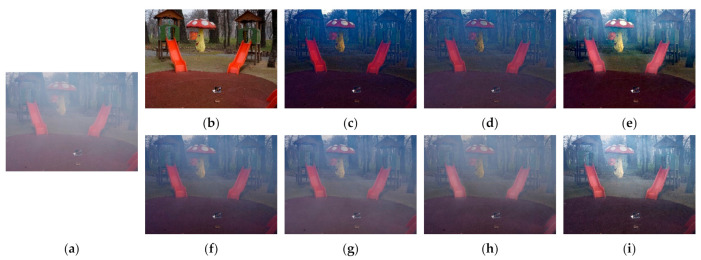
Comparison of dehazing results from O-HAZE dataset: (**a**) hazy image, (**b**) ground truth, (**c**) He et al., (**d**) Meng et al., (**e**) Berman et al., (**f**) Zhu et al., (**g**) Ren et al., (**h**) Cai et al., and (**i**) proposed.

**Figure 14:**
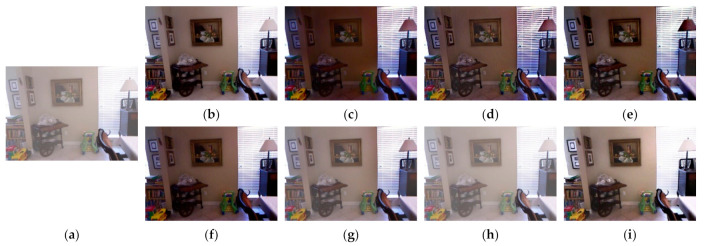
Comparison of dehazing results from SOTS indoor dataset: (**a**) hazy image, (**b**) ground truth, (**c**) He et al., (**d**) Meng et al., (**e**) Berman et al., (**f**) Zhu et al., (**g**) Ren et al., (**h**) Cai et al., and (**i**) proposed.

**Figure 15:**
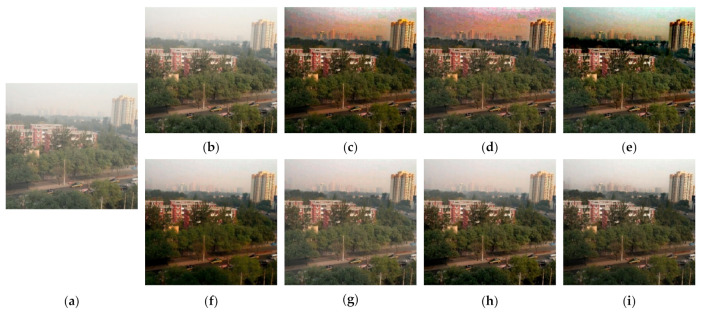
Comparison of dehazing results from SOTS outdoor dataset: (**a**) hazy image, (**b**) ground truth, (**c**) He et al., (**d**) Meng et al., (**e**) Berman et al., (**f**) Zhu et al., (**g**) Ren et al., (**h**) Cai et al., and (**i**) proposed.

**Figure 16:**
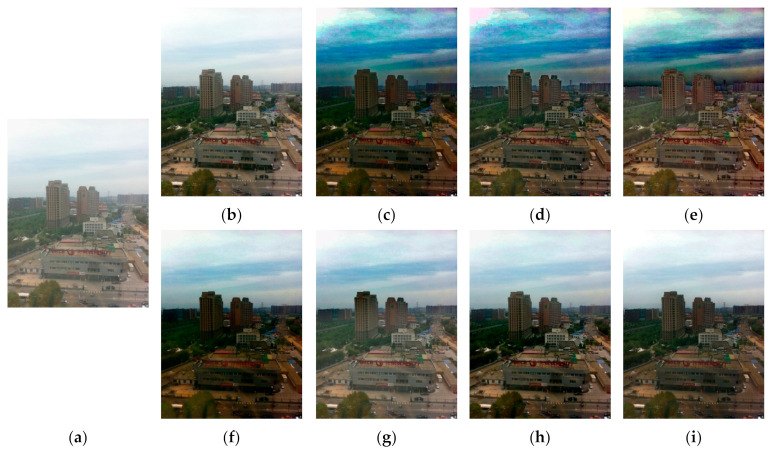
Comparison of dehazing results from HSTS dataset: (**a**) hazy image, (**b**) ground truth, (**c**) He et al., (**d**) Meng et al., (**e**) Berman et al., (**f**) Zhu et al., (**g**) Ren et al., (**h**) Cai et al., and (**i**) proposed.

Figure 12, Figure 13, Figure 14, Figure 15 and Figure 16 show detailed comparisons of different methods using the I-HAZE, O-HAZE, SOTS indoor, SOTS outdoor, and HSTS datasets, respectively. He et al. and Meng et al. darkened the image and caused distortions in bright areas such as the sky. Berman et al. removed too much haze, causing color distortion and saturation. Zhu et al. is unstable in terms performance and sometimes result in more or less hazy residues and in some cases dimmed images. Ren et al. and Cai et al. do not completely eliminate the haze. Ren et al. causes color distortions in several images. On the other hand, the proposed method recovers the closest image to the ground truth image, and the visual performance was much better.

#### 4.2.2. Qualitative Comparison of Hazy Image without Ground Truth

**Figure 17:**
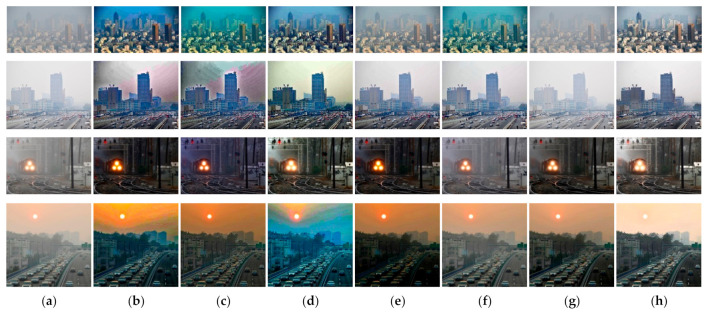
Comparison of dehazing results from real world image: (**a**) hazy image, (**b**) He et al., (**c**) Meng et al., (**d**) Berman et al., (**e**) Zhu et al., (**f**) Ren et al., (**g**) Cai et al., and (**h**) proposed.

**Figure 18:**
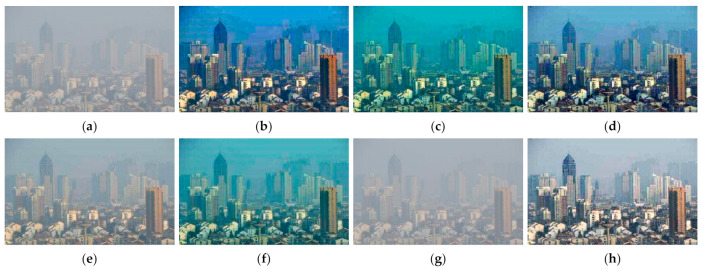
Comparison of dehazing results from real world image: (**a**) hazy image, (**b**) He et al., (**c**) Meng et al., (**d**) Berman et al., (**e**) Zhu et al., (**f**) Ren et al., (**g**) Cai et al., and (**h**) proposed.

**Figure 19:**
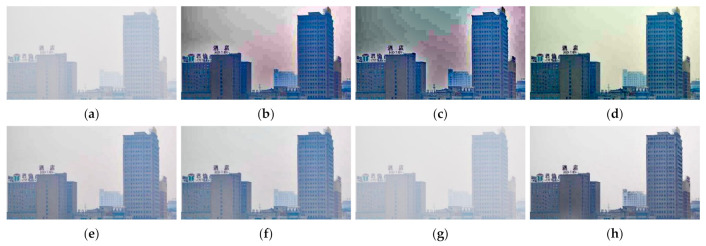
Comparison of dehazing results from real world image: (**a**) hazy image, (**b**) He et al., (**c**) Meng et al., (**d**) Berman et al., (**e**) Zhu et al., (**f**) Ren et al., (**g**) Cai et al., and (**h**) proposed.

**Figure 20:**
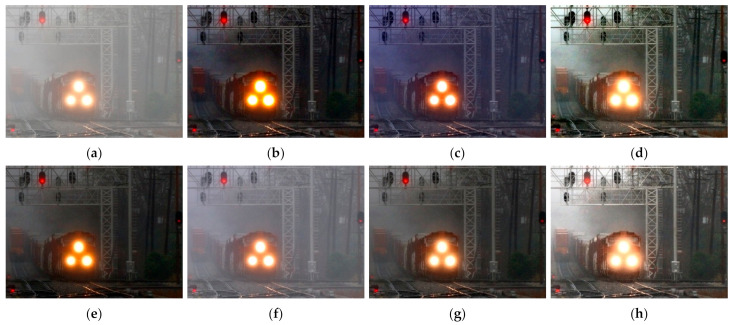
Comparison of dehazing results from real world image: (**a**) hazy image, (**b**) He et al., (**c**) Meng et al., (**d**) Berman et al., (**e**) Zhu et al., (**f**) Ren et al., (**g**) Cai et al., and (**h**) proposed.

**Figure 21:**
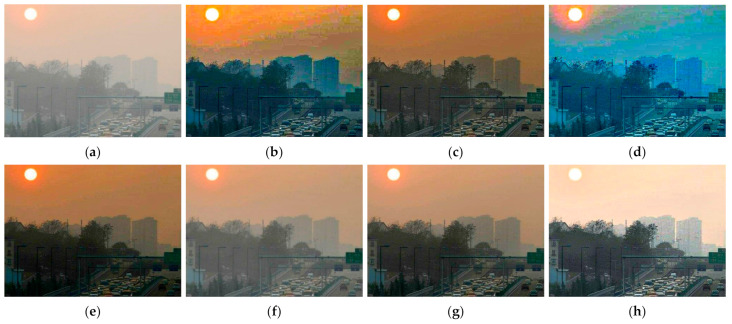
Comparison of dehazing results from real world image: (**a**) hazy image, (**b**) He et al., (**c**) Meng et al., (**d**) Berman et al., (**e**) Zhu et al., (**f**) Ren et al., (**g**) Cai et al., and (**h**) proposed.

Figure 17 compares dehazing results of real images. Figure 18, Figure 19, Figure 20 and Figure 21 show parts of each of Figure 17 in order to show the differences more clearly. As observed in Figure 18, Figure 19, Figure 20 and Figure 21, the proposed method outperforms the state of-the art haze removal methods in terms of the amount of haze removal and also without producing undesirable artifacts in flat, bright, and hazy regions. He et al., Meng et al., and Berman et al. produced artifacts in the sky area, and haze partially remained. Compared to the above methods, Ren et al. and Cai et al. showed stable results, but haze still remained in the far area, and there is some color distortion in the sky area. Zhu et al. dimmed the image and left haze in the results. These results prove the effectiveness of the proposed method in real cases.

### 4.3. Limitations of Proposed Method

**Figure 22:**
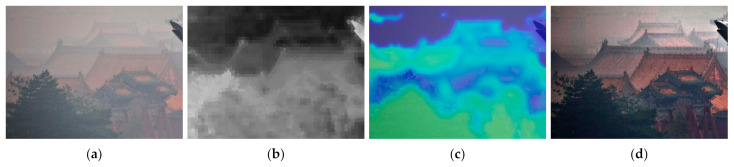
Example of noise boosting problem: (**a**) input image, (**b**) entropy of input image, (**c**) enhancement map, and (**d**) result image.

**Figure 23:**
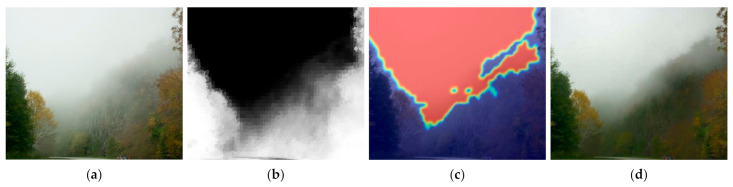
Example of haze remaining problem: (**a**) input image, (**b**) entropy of input image, (**c**) prevention map, and (**d**) result image.

The effectiveness of the proposed method for haze removal is shown in Section 4.1 and Section 4.2. In this section, the limitations of the proposed method are presented. Figure 22 shows the case where noise exists in the texture areas. In this case, the texture region is considered as a visibility enhancement region; thus, a large $\omega_{p}\left(x\right)$ value is applied to remove haze while amplifying noise. Figure 23 shows the case of misclassifying the texture as a flat area. Due to dense haze and weak textures, the entropy of distant mountains is relatively low, which causes this region to be misclassified as a flat region. As a result, a small $\omega_{p}\left(x\right)$ value is applied, resulting in relatively little haze removal.

### 4.4. Computational Complexity

Table 3 shows the average running time in order to show the computational efficiency of the proposed method. Experiments were performed by considering all methods at different resolutions (640 × 480, 1024 × 768, 1280 × 720, and 1920 × 1080) on a PC equipped with 2.8 Ghz Intel Core i7 and 16 GB RAM. The proposed method is implemented in C++, and the other methods are implemented in Matlab. The differences in implementation cannot result in a fair comparison, but the effectiveness of the proposed method is clearly visible.

## 5. Conclusions

In this paper, a method to recover images from single hazy image was presented. To this end, dehazing was performed by combining complementary *DCP* and *BCP*. A patch-based robust atmospheric light estimation has been proposed for dividing the image into regions to which the *DCP* assumption and the *BCP* assumption are applied. In addition to brightness, saturation and contrast were taken into account when estimating atmospheric light in order to avoid erroneous selection of white or bright landscape objects such as the atmosphere. A region adaptive haze control parameter was introduced to prevent artifacts in bright and hazy areas of low detail and to improve haze removal in high detail hazy areas. Shannon’s entropy was used to compute texture/flat probabilities, then the prevention and enhancement weights for each superpixel were calculated. The performance of the proposed method was evaluated by using qualitative and quantitative analysis of synthetic images and real-world images. Experiments confirmed that the proposed method effectively prevent the artifacts of the flat and bright area while effectively removing haze. The results of the proposed algorithm showed better performance than the conventional methods in both quantitative and qualitative criteria.

## Figures and Tables

**Table 1:** Quantitative comparison of different methods with ground truth.

Dataset	Metric	He et al.	Meng et al.	Berman et al.	Zhu et al.	Ren et al.	Cai et al.	Proposed
I-Haze (35 samples)	PSNR	11.56	13.11	13.87	14	**16.47**	14.55	16.26
	SSIM	0.42	0.51	0.51	0.5	0.59	0.53	**0.61**
O-Haze (45 samples)	PSNR	14.72	15.49	15.15	15.49	**16.76**	15.21	15.78
	SSIM	0.38	0.42	0.46	0.37	0.4	0.42	**0.49**
SOTS-Indoor (500 samples)	PSNR	16.81	17.05	17.29	18.98	17.13	11.97	**19.42**
	SSIM	0.82	0.79	0.75	0.85	0.81	0.68	**0.86**
SOTS-Outdoor (500 samples)	PSNR	14.81	15.57	18.08	18.25	19.61	**22.92**	20.96
	SSIM	0.7549	0.783	0.8026	0.7867	0.8633	0.8886	**0.8966**
HSTS (10 samples)	PSNR	15.09	15.16	17.63	19.84	18.67	**24.48**	22.36
	SSIM	0.7656	0.7414	0.7933	0.8157	0.8174	**0.9216**	0.9

**Table 2:** Quantitative comparison of different methods with real-world images.

Metric	Input	He et al.	Meng et al.	Berman et al.	Zhu et al.	Ren et al.	Cai et al.	Proposed
NIQE	3.19	3.1	3.22	3.49	3.11	3.15	3.1	**2.95**
BRISQUE	32.24	28.67	25.91	29.72	30.65	30.11	30.52	**24.53**
PIQE	41.15	40.74	40.35	44.75	41.16	41.2	41.3	**36.02**
IE	6.97	6.98	7.03	7.35	7.11	7.24	7.14	**7.4**

**Table 3:** Computational complexity comparisons.

Image Size	He et al.	Meng et al.	Berman et al.	Zhu et al.	Ren et al.	Cai et al.	Proposed
640 × 480	1.359776	2.986463	2.637870	0.690927	1.854696	1.619289	0.344
1024 × 768	3.535564	4.032741	4.140068	1.358614	2.587211	3.576306	0.535
1280 × 720	4.097585	3.678053	4.488762	1.749579	2.906746	4.373431	0.547
1920 × 1080	9.534287	6.824747	8.149385	3.182058	6.432193	9.872256	0.636

## Data Availability

Data sharing no applicable.
